# Loop Entropy Assists Tertiary Order: Loopy Stabilization of Stacking Motifs

**DOI:** 10.3390/e13111958

**Published:** 2011-11-24

**Authors:** Daniel P. Aalberts

**Affiliations:** Physics Department, Williams College, Williamstown, MA 01267, USA; Tel.: +1-413-597-3520; Fax: +1-413-597-4116

**Keywords:** RNA, coaxial stacking, tertiary structure, secondary structure, multibranch loops, Hairpin loops, polymer simulations

## Abstract

The free energy of an RNA fold is a combination of favorable base pairing and stacking interactions competing with entropic costs of forming loops. Here we show how loop entropy, surprisingly, can promote tertiary order. A general formula for the free energy of forming multibranch and other RNA loops is derived with a polymer-physics based theory. We also derive a formula for the free energy of coaxial stacking in the context of a loop. Simulations support the analytic formulas. The effects of stacking of unpaired bases are also studied with simulations.

## 1. Introduction

The classic competition of low energy versus high entropy states is dramatically demonstrated in most phase transitions; for example, in water, electrical attraction stabilizes the liquid state while there are vastly more microstates in the vapor state. In this letter, we describe a curious counterexample to this competition in which the ordered tertiary structure of a biopolymer has not only the lower energy, as expected, but also, surprisingly, the higher entropy as well. The key for this to occur is that the structural elements must be part of a loop. We will show, for example, how loop entropy contributes to the stabilization of the tRNA fold.

The secondary structure of RNA is created by the pairing of complementary bases to form double-helical regions. Single-stranded regions, made up of unpaired bases, connect the helices. The organization of these helices in space constitutes the tertiary fold. RNA helices often adopt a coaxially-stacked geometry reflecting beneficial base-stacking interactions. Let us now consider coaxial stacking of two helices in two related scenarios.

**Figure 1:**
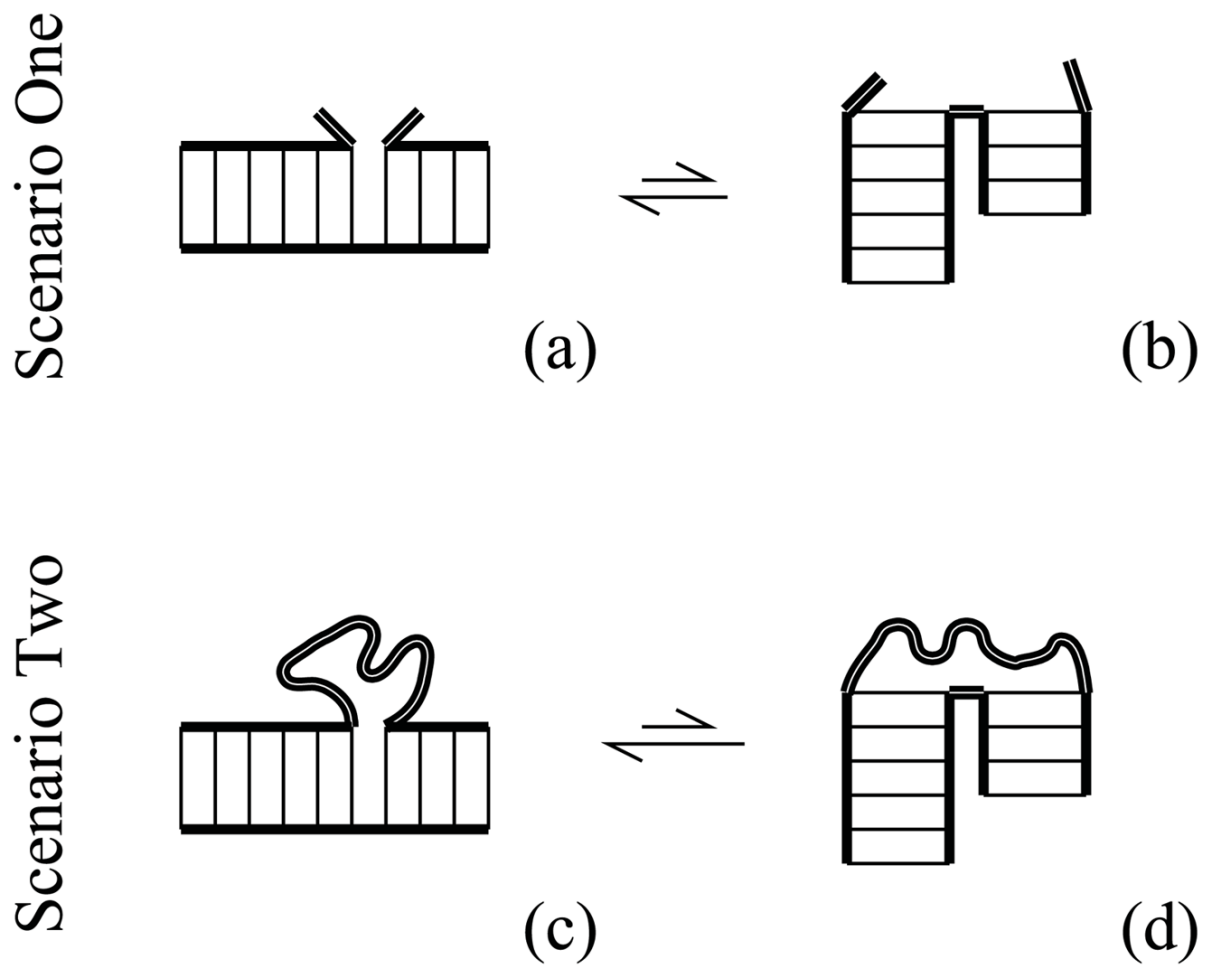
Scenario One, adjacent helices: (a) the low enthalpy state with coaxially-stacked helices; (b) the high entropy state has unstacked helices and a flexible hinge (thick double lines). Scenario Two, adjacent helices are now part of a closed loop; (c) the greatest loop entropy occurs in the low-enthalpy coaxially-stacked state; (d) unstacked helices stretch the loop, so they have less loop entropy than (c).

In Scenario One of Figure 1, the RNA consists of two helices joined by a single backbone link. The stacked state (a) has low enthalpy, while unstacked state (b) has greater entropy. The free energy difference between these states Δ*G*_ab_ = Δ*H*_ab_ − *T*Δ*S*_ab_, is estimated in the Turner rules [1] to provide about one to three kcal/mol stability just like other nearest-neighbor basepair stacks.

In Scenario Two of Figure 1, the RNA consists of two helices joined by a chain on one side and a single backbone link on the other side. Again these helices will adopt an equilibrium between stacked (c) or unstacked (d) configurations. The stacked configuration (c) is again the one with lower enthalpy, but here the number of chain microstates must now be included in calculating the total entropy. Chain entropy promotes the stacked state, as we shall see.

There are many more chain configurations when the chain ends are near [the stacked state, (c)] than stretched [any of the unstacked states, (d)]. Rubber elasticity is explained by this well-known property. The net entropic difference is always reduced in a loop context (Scenario Two) relative to the open case (Scenario One), Δ*S*_dc_ < Δ*S*_ba_. Thus, a loop context always provides additional stability to ordered, stacked states.

**Figure 2:**
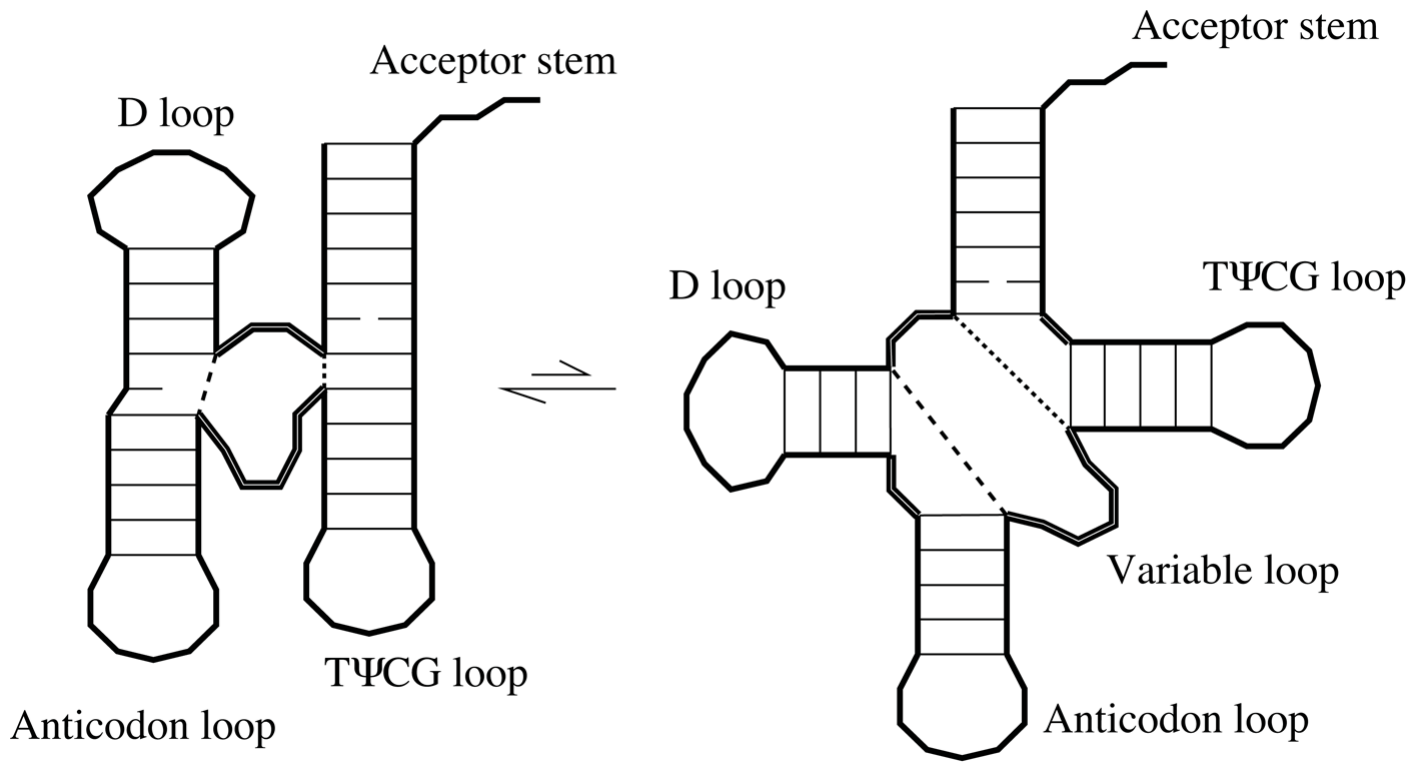
The secondary structure of a tRNA includes four base-paired stems meeting at a multibranch loop. The flexible parts of a multibranch loop (thick double lines) might adopt many configurational orientations. Two pairs of helices can coaxially stack and, doubling what we saw in Figure 1, helix stacking increases the loop entropy because it brings the chain ends close together (note the change in lengths of the dashed and dotted lines). The crystal structure of tRNA exhibits the double stacking of the stacked configuration.

Let us now see how loop entropy can contribute to tertiary order in an important molecule. In the famous three-leaf clover fold of tRNA, four helices meet in the central multibranch loop, see Figure 2. Scenario Two of Figure 1 is played out twice in tRNA’s multibranch loop as two pairs of helices stack. If the acceptor and TΨCG stems stack, the opposite edges are brought near (dotted lines in Figure 2); the chain (the remainder of the multibranch loop, including single-stranded segments and the D and anticodon stems) has more microstates when the ends are close. Likewise, if the anticodon and D stems stack with an intervening mismatch, then there is less distance to traverse (compare dashed lines in Figure 2). In the next section, we will estimate an additional loop-entropic stabilization of −0.5 kcal/mol for each stack as *M*_eff_ goes from four to two, or from two to zero.

In [2] we introduced the enhanced two-length freely jointed chain (FJC2) model. FJC2 captures the reality that the two length scales that comprise RNA loops are quite different. The length of single-stranded segments *a* = 6.2 Å, and the helix diameter *b* = 15 Å, are obtained by measuring ° between backbone 4′ carbons in PDB files. (The disparity in these lengths is indicated in the figures.)

The FJC2 model [2] contributes to a grand history of studying loops in biopolymers [3–12]. By introducing the two length scales, a universal formula for the free energy of loops with different number of helices (from hairpins to multibranch loops) can be derived from polymer physics. In [8], for example, simulations were fit to a generalized Jacobson-Stockmayer formula finding different curvatures at different numbers of helices; however, we will show below that our formula provides a universally good description of simulations with many fewer parameters.

## 2. Methods

In the previous section, we aimed for an intuitive description of coaxial stacking in RNA loops. In this section, we will take a more mathematical approach and derive multiplicities in three dimensions, with two length scales *a* and *b*. We begin with the “phantom chain” or “random walk” approximation of the FJC2 model, then amend those formulas for self-avoidance, and finally test them with simulations.

### 2.1. Phantom-Chain Approximation

First we give some preliminaries. In the FJC2, segment’s randomly distributed unit vectors obey 〈**n̂***_i_* 〉 = 0 and 〈**n̂***_i_* · **n̂***_j_*〉 = *δ_ij_*, and segment lengths *r_i_* ∈ {*a*, *b*}. The total end-to-end separation is **R** = Σ*_i_ r_i_***n̂***_i_*, whose second moment is,

$$\langle R^{2}\rangle =\sum_{i,j}r_{i}r_{j}\langle {\hat{n}}_{i}\cdot {\hat{n}}_{j}\rangle =\sum_{i}r_{i}^{2}=Na^{2}+Mb^{2}$$

if there are *N* links of length *a* and *M* links of length *b*. This reduces to the familiar FJC result if there is only one segment length type.

For the derivation, it is convenient to introduce *z* to represent the number of directions available to the link at each step. Recent work [7–10] have enumerated configurations with discrete number of possibilities at each step, many based on a virtual bond representation of the backbone [13,14].

For an unconstrained, open chain with *N* links of length *a* and *M* links of length *b*, each of which can point in *z* directions, the total number of microstates is

$$\Omega_{\text{open}}=z^{N+M}$$

Only a fraction of walks make a loop. Loops are formed when a polymer walk returns near its starting point so the probability of making a loop scales like

$$p_{\text{loop}}\sim \Delta V/{\langle R^{2}(N,M)\rangle }^{3/2}$$

where Δ*V* is a measure of nearness and 〈*R*^2^(*N*, *M*)〉^1/2^ is the typical chain extension.

The number of loop microstates is this fraction of the total

$$\Omega_{\text{loop}}(N,M)\sim z^{N+M}\frac{\Delta V}{{\langle R^{2}(N,M)\rangle }^{3/2}}$$

This means the free energy to initiate a loop is

$$\begin{array}{l} \Delta G_{\text{loop}}(N,M)=-\mathrm{kT}\ln(\Omega_{\text{loop}}/\Omega_{\text{open}})\\ =\frac{3}{2}\mathrm{kT}\ln(Na^{2}+Mb^{2})+C \end{array}$$

where the constant *C* accommodates dimensionful factors.

Let us next derive the effect of helix stacking in the phantom-chain approximation. If helices are stacked, then the second *b* segment’s orientation is specified to be opposite of the first, and the effective loop length is reduced by two *b* segments. Since *b*^2^ ≫ *a*^2^, the effect can be dramatic. The number of stacked microstates is

$$\Omega_{\text{stack}}\sim \frac{\Delta V}{{\langle R^{2}(N,M-2)\rangle }^{3/2}}z^{N+M-1}$$

So the ratio

$$\frac{\Omega_{\text{loop}}(N,M)}{\Omega_{\text{stack}}(N,M)}=\frac{z\cdot {\langle R^{2}(N,M-2)\rangle }^{3/2}}{{\langle R^{2}(N,M)\rangle }^{3/2}}$$

is always smaller than *z*, which is the value without a loop (Scenario One in Figure 1). This means the loop always provides entropic stabilization to helix stacking. The benefit of being in a loop is greater when *b*^2^/*a*^2^ is large or *N* is small.

Stacking produces a net free energy change of

(1) $$\begin{array}{l} \Delta G_{\text{stack}}=(-\varepsilon -\mathrm{kT}\ln\Omega_{\text{stack}})-(-\mathrm{kT}\ln\Omega_{\text{loop}})\\ =-\varepsilon +\mathrm{kT}\ln z-\frac{3}{2}\mathrm{kT}\ln\frac{\langle R^{2}(N,M)\rangle }{\langle R^{2}(N,M-2)\rangle }\\ =-\varepsilon +\mathrm{kT}\ln z-\frac{3}{2}\mathrm{kT}\ln\frac{Na^{2}+Mb^{2}}{Na^{2}+(M-2)b^{2}} \end{array}$$

The first terms are the expected (Scenario One) coaxial stacking free energy −*ε* +*kT* ln *z*, while the final term is the additional stabilization from the loop entropy.

### 2.2. Self-Avoiding Chain Approximation

The formulas we have just derived in the phantom-chain approximation can be revised to include self-avoidance. A self-avoiding chain is somewhat extended,

$$R_{\text{sa}}^{2}={\langle R^{2}(N,M)\rangle }_{\text{sa}}=N^{6/5}a^{2}+M^{6/5}b^{2}$$

which, if *M* = 0, reduces to the familiar Flory theory result *R_F_* = *N*^3/5^*a*.

For self-avoiding chains, when *r* ≪*R*_sa_, the probability density scales like [15]:

$$p_{\text{sa}}(r)\;dr\sim {\left(\frac{r}{R_{\text{sa}}}\right)}^{5/18}\frac{4\pi r^{2}\;dr}{R_{\text{sa}}^{3}}$$

So, after integrating over a small volume, one finds the probability of making a loop scales like

$$p_{\text{loop}}\sim R_{\text{sa}}^{-5/18-3}\sim {\langle R^{2}\rangle }_{\text{sa}}^{-59/36}$$

and the number of self-avoiding loop microstates goes like

$$\Omega_{\text{loop}}(N,M)\sim \frac{z^{N+M}}{{\langle R^{2}(N,M)\rangle }_{\text{sa}}^{59/36}}$$

This means, with self-avoidance, the free energy to initiate a loop becomes

(2) $$\begin{array}{l} \Delta G_{\text{loop}}(N,M)=-\mathrm{kT}\ln(\Omega_{\text{loop}}/\Omega_{\text{open}})\\ =\frac{59}{36}\mathrm{kT}\ln(N^{6/5}a^{2}+M^{6/5}b^{2})+C \end{array}$$

Although Equation (2) is derived assuming many segments, we will show below with simulations that it continues to be a good description even for loops with relatively few segments. The functional form of Equation (2) can also be generalized to describe chains with single-stranded base stacking interactions.

Even with self-avoidance, stacking of helices specifies the direction of a *b* segment and the remainder of the loop has two fewer *b* segments than before, so the free energy to stack two helices becomes

(3) $$\begin{array}{l} \Delta G_{\text{stack}}=(-\varepsilon -\mathrm{kT}\ln\Omega_{\text{stack}})-(-\mathrm{kT}\ln\Omega_{\text{loop}})\\ =-\varepsilon +\mathrm{kT}\ln z-\frac{59}{36}\mathrm{kT}\ln\frac{N^{6/5}a^{2}+M^{6/5}b^{2}}{N^{6/5}a^{2}+{\left(M-2\right)}^{6/5}b^{2}} \end{array}$$

Again, the first terms are the expected free energy change for coaxial stacking and the last term is the additional entropic stabilization contribution of the loop, now including self-avoidance.

Returning to our tRNA example in Figure 2, after two pairs of helices stack, *M* = 4 becomes *M*_eff_ = 0. With *N* ≈ 12 for tRNA’s multibranch loop, we find about −1 kcal/mol in entropic stabilization. The entropic stabilization supplements the other free energy contributions of MFOLD, a widely used program to predict optimal and suboptimal RNA folds [19]. In [2], we implemented our formula for RNA loops that includes this additional entropic stabilization mechanism and evaluated on a test set of 569 tRNA, 309 5S-RNA, 369 SRP, 66 hammerhead and 41 *cis*-regulatory sequences. We found [2] that MFOLD’s prediction accuracy improves substantially.

### 2.3. FJC2 Simulations, Including Base-Stacking in Loops

Equation (2) describes the free energies of loop formation for chains of different composition. As in [2], we test its validity by performing Monte Carlo simulations on polymers, modeled as volume-excluding beads of radius *r* = 2.4 Å separated by *a* = 6.2Å or *b* = 15Å (intermediate beads were placed 6.2 Å from each end of the *b* segments to ensure self-avoidance). More than 10^8^ self-avoiding random walks were constructed containing (*N* − 1) steps of length *a* and *M* steps of length *b*. Walks whose end-to-end separation was in the range 5.7Å to 6.7 Å were considered to make a loop; since that separation is approximately *a*, this represents an (*N*, *M*) loop.

**Figure 3:**
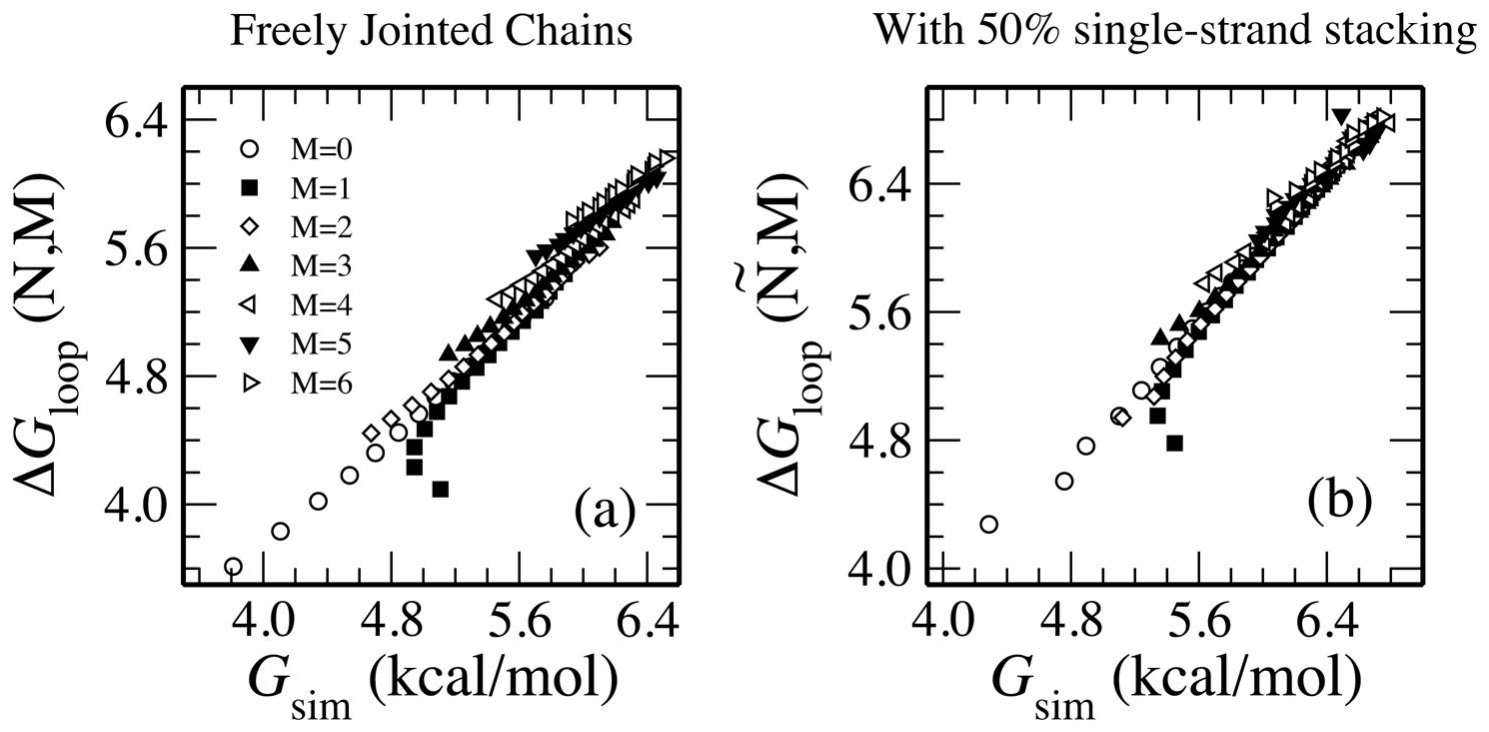
Although our formulas for the free energy of loop formation were derived for long chains, we find the theory gives similar values to simulations for long or short chains and with many or few helices. Values for *N* ≤ 20 and *M* ≤ 6 are plotted. In (a), simulations with freely-jointed segments are compared with Equation (2). In (b), with 50% probability the next *a* segment continues in the same direction (representing bases stacked); the other 50% are freely jointed. This compares favorably with Equation (4).

The free energy of the simulation is *G*_sim_(*N*, *M*) = −*kT* ln[Ω_loop_(*N*, *M*)/Ω(*N*, *M*)], where Ω(*N*, *M*) is the number of self-avoiding walks and Ω_loop_(*N*, *M*) is the number which make loops. In Figure 3(a), we see that Equation (2) is a universally very good fit for simulations over a range of (*N*, *M*) values, including short chains.

To investigate the effect of base stacking in the single-stranded regions, we performed additional simulations. The persistence length within single-stranded regions is estimated to be about two segments [16,17] This corresponds to a Δ*G* = 0 for stacking of bases [6,18], or equal probability of stacking or not stacking at each step. Simulations were modified so that half of added *a* segments would continue in the same direction as the previous one (stacked) and that half would be freely jointed. To account for stacking, we generalize Equation (2) with an effective segment length *ã* which can be longer than *a* and an effective number of segments *Ñ* = *Na*/*ã*.

(4) $$\Delta G_{\text{loop}}(\tilde{N},M)=\frac{59}{36}\mathrm{kT}\ln({\tilde{N}}^{6/5}{\tilde{a}}^{2}+M^{6/5}b^{2})+C$$

In Figure 3(b) we find excellent agreement to simulations using *ã* = 10Å.

## 3. Conclusions

In summary, we have derived how loop entropy favors ordered tertiary structures in RNA. Equations (1) and (3) are our formulas describing “Phantom” and “Self-Avoiding” cases, respectively. The loop-entropy stabilization comes in addition to the more expected stabilization of coaxial stacking seen in Scenario One. Including the effect of helix stacking on loop stabilization improves the prediction accuracy of MFOLD, underscoring its importance in the folding problem.

Our simulations indicate the formulas continue to work even with relatively short chains and even when typical stacking of the unpaired bases is included.

There are other examples of entropic-driven order called “depletion forces” or “molecular crowding” [20,21] which are observed between colloidal particles and surfaces in the presence of small particles. The mechanism is that small particles gain volume when large particles crowd together. Entropically-driven order is also known in liquid crystal systems where increasing concentration first aligns the axes of long molecules and then layers them in planes (for a review see for example [22]). We believe ours is the first description of the entropy of polymer loops contributing favorably to tertiary ordering.

Predicting the secondary and tertiary folds of macromolecules is one of the grand challenges of computational biology, and our loop entropy stabilization principle may have broad application to the folding problem.
